# Efficacy and Safety of Beclomethasone Dipropionate versus 5-Aminosalicylic Acid in the Treatment of Ulcerative Colitis: A Systematic Review and Meta-Analysis

**DOI:** 10.1371/journal.pone.0160500

**Published:** 2016-08-08

**Authors:** Xin Zhao, Nan Li, YiMing Ren, Tao Ma, ChunLi Wang, Jun Wang, ShengYi You

**Affiliations:** 1 Department of General Surgery, Tianjin Medical University General Hospital, Tianjin, China; 2 Department of Orthopedics, Tianjin Medical University General Hospital, Tianjin, China; University Hospital Llandough, UNITED KINGDOM

## Abstract

**Background:**

Ulcerative colitis (UC) is a chronic and remitting inflammatory disease that is characterized by chronic idiopathic inflammation of the colon and bloody diarrhea. Currently drug treatment is the main intervention for patients with mild to moderate UC. Mesalazine (5-ASA) and beclomethasone dipropionate (BDP) have been widely used for the treatment of UC and have yielded satisfactory results. This study compared the effectiveness of 5-ASA and BDP in the treatment of UC.

**Methods:**

The PubMed, Medline, SinoMed, Embase, and Cochrane Librinary databases were searched for eligible studies. Data were extracted by two of the coauthors independently and were analyzed using RevMan statistical software, version 5.3. Weighted mean differences (WMDs), odds ratios (ORs), and 95% confidence intervals (CIs) were calculated. Cochrane Collaboration’s Risk of Bias Tool was used to assess the risk of bias.

**Results:**

Seven randomized controlled trials that compared BDP with 5-ASA in treating UC were identified as eligible. The methodological quality of the trials ranged from low to moderate. A pooled analysis of effectiveness based on the Disease Activity Index (DAI) or other assessment method after treatment revealed that in the treatment of UC, there are no obvious differences between BDP and 5-ASA in inducing remission and clinical improvement (OR = 0.76, 95% CI = 0.56–1.03, P = 0.08). The total numbers of adverse events associated with BDP and 5-ASA treatments for UC were similar (OR = 1.21, 95% CI = 0.71–2.09, P = 0.48). The safety profiles for these two drugs are good. According to subgroup-analysis, we found no obvious differences of clinical efficacy between BDP and 5-ASA no matter oral or enema administration was used in the treatment of UC. A sensitivity analysis demonstrated the stability of the pooled results.

**Conclusion:**

During induction treatment of mild to moderate UC, there is no obvious difference between the two groups with respect to remission and clinical improvement. Given that the upper confidence limit for the OR barely exceeds 1.0 and that the p-value is close to 0.05 for this primary efficacy outcome as well as that the horizontal block lies to the left of the vertical line, it indicates that the clinical efficacy of BDP may be better than 5-ASA. However, taking into account that BDP has the risk of hypothalamic-pituitary-adrenal axis (HPA) suppression, 5-ASA has a potential advantage of safety in the treatment of mild to moderate UC.

## Introduction

Ulcerative colitis (UC) is an idiopathic condition characterized by intermittent episodes of chronic inflammatory bowel disease that has become a worldwide disease [1]. Studies have suggested that UC may be a disorder of the immune system caused by changes in the environment [2,3]. Related studies have shown that approximately 600,000 people in the United States are affected by the disease [4]. UC carries a high burden of morbidity because of its long duration and recurrent attacks. Patients often experience intermittent episodes of disease activity that can seriously affect their health and quality of life [5]. Currently, the main treatment methods for UC are drug therapy and surgical treatment. The only major indications for surgical treatment are life-threatening acute severe UC, ineffectiveness of drug treatment, and recurrence and serious impact on daily living in patients with chronic UC. It was noted in the 2010 American Gastroenterology Practice Guidelines that topical or oral 5-aminosalicylic acid (5-ASA) and corticosteroids can be recommended as treatments for mild-to-moderate UC [6].

5-ASA, also known as mesalazine, has been recommended as a first-line drug for UC treatment [7]. So far, the mechanism of 5-ASA is not completely understood, and Rousseaux C et al. [8] found that 5-ASA may exert anti-inflammatory effects through inhibition of leukotriene and prostaglandin E as well as production of free radicals. Glucocorticosteroid drugs were first used over 60 years ago, and the first controlled trial demonstrating their efficacy in the treatment of UC was conducted in the 1950s [9,10]. Glucocorticoids reduce inflammation by down-regulating the transcription of genes involved in pro-inflammatory cytokines, inhibition of inflammatory tissue adhesion molecule expression, and activation of immune cells [11]. However, glucocorticosteroid drug use is limited by the frequent and sometimes severe side effects of such drugs. Steroid-related side effects include metabolic, dermatological, gastrointestinal, musculoskeletal (ranging from osteopenia to osteoporosis), and central nervous system effects, as well as hypertension, hypothalamic-pituitary-adrenal axis suppression, and infections [12]. Thus, a second generation of corticosteroids with fewer and less severe systemic effects has been developed. Beclomethasone dipropionate (BDP) is a second-generation corticosteroid with a first-pass effect and low systemic bio-availability characteristics. It has strong anti-inflammatory effects and could largely reduce the long-term complications associated with traditional corticosteroids and inhibition of the hypothalamus—pituitary—adrenal axis [13]. A study has shown that BDP can effectively reduce the incidence of long-term complications [14]. The anti-inflammatory activity of BDP in the treatment of UC has been demonstrated in several clinical trials [15,16]. Some studies have shown that BDP can be considered a viable alternative to topical 5-ASA therapy with significantly lower inhibition of plasma cortisol levels [17,18]. However, the latest published meta-analysis by Manguso and Balzano indicated that BDP and 5-ASA have the same efficacy in inducing clinical remission of UC [19]. Whether BDP is more efficacious in the treatment of UC should be examined. To conduct an updated study and to provide more evidence for clinical decision-making, we collected published and unpublished studies covering randomized controlled trials (RCTs) of BDP vs. 5-ASA in the treatment of UC and performed a meta-analysis to evaluate its efficacy and safety.

## Materials and Methods

### Data sources and search strategy

A computerized search of the PubMed, Medline, SinoMed, Embase, and Cochrane Library databases was conducted from inception to June 2015 (XZ, NL, TM). The databases were queried for eligible literature using combinations of the following keywords: Beclomethasone dipropionate or BDP, 5-aminosalicylic acid or 5-ASA, mesalazine or mesalamine, and ulcerative colitis. The search was limited to human subjects. The titles and abstracts of potentially relevant studies identified by the computerized search were reviewed. Furthermore, the reference lists of the retrieved studies were also assessed to identify additional relevant articles. In addition, we reviewed abstracts from a conference of the major worldwide Inflammatory Bowel Disease (IBD) meeting.

### Inclusion and exclusion criteria

The inclusion criteria were the following: (1) the study included outpatients of either sex, aged 18 to 70 years, with a clinical, endoscopic, and histological diagnosis of mild-to-moderate ulcerative colitis (proctitis, proctosigmoiditis, and left-sided colitis); (2) outcomes at least included Disease Activity Index (DAI) [20] scores or other indexes of clinical improvement; (3) patients were treated with either BDP or 5-ASA; (4) the study covered published clinical RCTs or retrospective, controlled studies; and (5) studies covering the same populations were represented by only the most eligible study.

The exclusion criteria used were as follows: (1) data description or sample information that was insufficiently clear; (2) inappropriate statistical methods; and (3) application of other drugs or therapies during the treatment.

### Assessment of quality and data extraction

Two coauthors (CLW, JW) independently reviewed all of the titles and abstracts of the searched papers. The full texts of potentially qualified papers that met the inclusion criteria were examined to determine whether they were indeed eligible. Extracted data included the characteristics of the eligible studies, such as period, country, study design, sample size, sex, mean age, disease location, evaluation method, number of interventions, and relevant outcomes. Advice was sought through discussion or from a third partner (SYY) to resolve inconsistent evaluations.

### Outcomes

The primary outcomes were remission rate and clinical improvement as assessed by the DAI or other similar scores in each study, and the secondary outcome measures were changes in single DAI parameters and the safety of the two drugs, such as drug-related adverse events (AEs) (e.g., morning plasma cortisol levels, monitoring of pituitary-adrenal insufficiency, laboratory parameters, and clinical signs). Major signs of pituitary-adrenal dysfunction include leg edema, Cushing-like syndrome, or newly diagnosed high blood pressure and diabetes. The following laboratory parameters were collected: white blood cell (WBC) count, red blood cell (RBC) count, hemoglobin, platelet count, and levels of plasma glucose, BUN, creatinine, alanine aminotransferase, aspartate aminotransferase, sodium, potassium, and magnesium.

### Risk of bias

The risks of bias for RCT studies were evaluated with the Cochrane Collaboration’s Risk of Bias Tool. Seven parameters were evaluated for each included study: random sequence generation, allocation concealment, blinding, incomplete outcome data, selective outcome reporting, and other risks. Items were judged as "low risk", "unclear risk", or "high risk".

### Statistical analysis

A meta-analysis was conducted using the software Review Manager 5.3 (Cochrane Collaboration, http://tech.cochrane.org/revman/download). For dichotomous outcomes, the odds ratio (OR) and 95% confidence interval (CI) were calculated, whereas for continuous outcomes, the standardized mean difference (SMD) and 95% CI were used. The SMD was preferred over the weighted mean difference because different measurement indexes that adopted different tools were used in the various studies. Raw data for each outcome were extracted and converted into individual 2×2tables (BDP vs. 5-ASA). The Cochrane Handbook's Q test and I^2^ statistic were used to determine the heterogeneity among the studies. If there was significant heterogeneity (P < 0.05, I^2^ > 50%), a random-effects model was used. Otherwise, fixed-effects models were applied if there was no significant heterogeneity (P ≥ 0.05, I^2^ ≤ 50%). Subgroup analyses based on the formulations of 5-ASA and BDP (oral vs. enema) were performed to compare the efficacies of the two drugs. In our meta-analysis, if the degree of heterogeneity was greater than the statistical test results, a sensitivity analysis was performed.

## Results

### Data extraction

**Fig 1:**
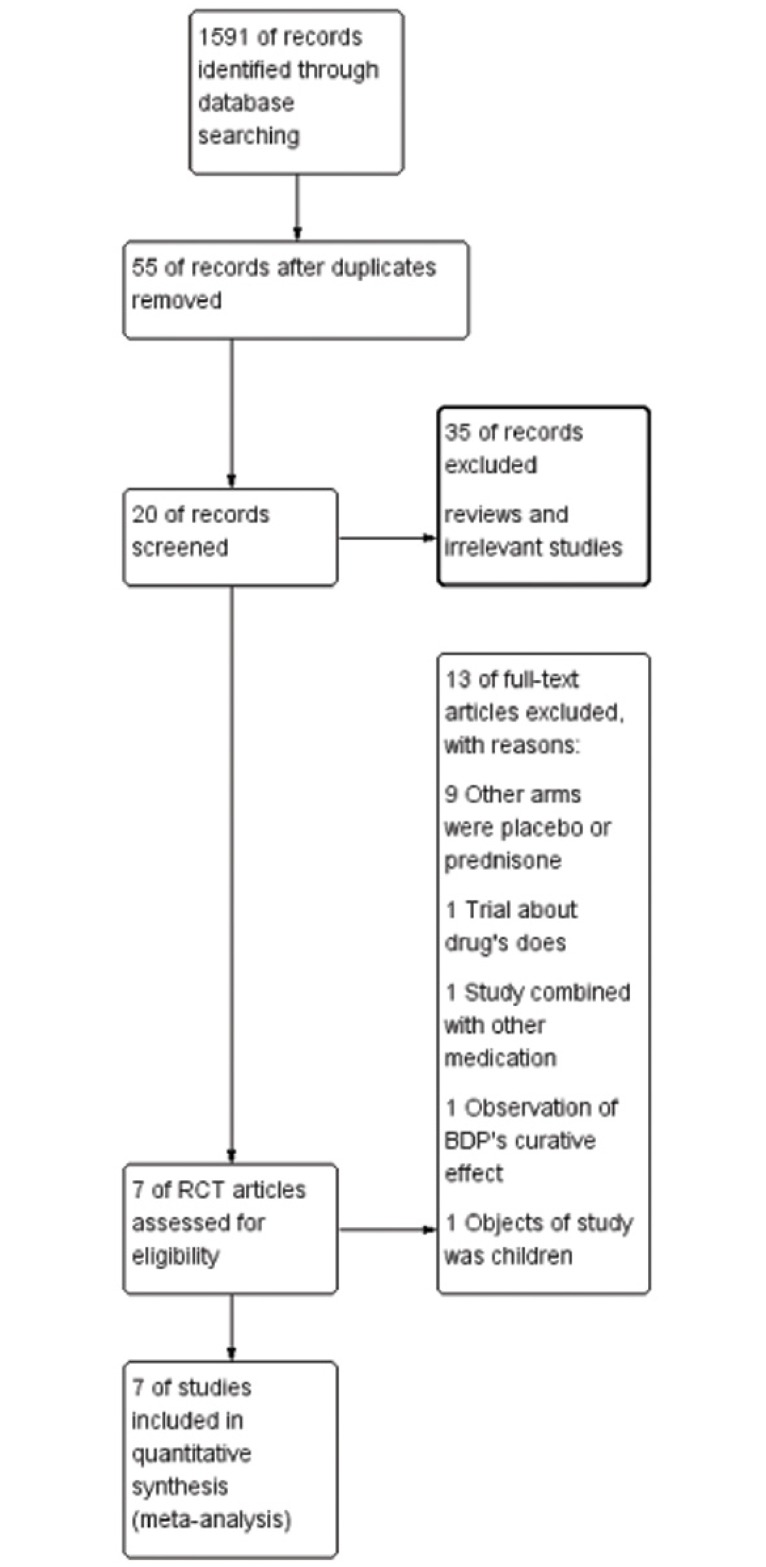
Flow diagram for selection of randomized controlled trials (RCTs) included in the meta-analysis.

Of the 1591 citations identified based on a study of the subject and summary of the literature, 1536 articles were excluded due to duplication. Fifty-five literatures were remained. Based on the title and abstracts review, 35 irrelevant articles were excluded. Twenty full text articles were assessed for eligibility. Finally, seven clinical studies satisfied the inclusion requirements [21–27]. A detailed study flow diagram is shown in Fig 1.

### Study characteristics

The characteristics of the included studies are presented in Table 1. The 7 assessed studies included six in English and one in Italian; useful information was translated when necessary. Among the seven eligible studies, five studies evaluated the curative effects of either BDP or 5-ASA in the treatment of UC, whereas two trials evaluated the two drugs in combination compared with single administration. We extracted relevant useful data to conduct our analyses. The majority of patients enrolled in these studies had standard clinical, endoscopic, and histological diagnoses of mild to moderate distal UC (proctitis, proctosigmoiditis, and left-sided colitis). The included studies were conducted from 1996 to 2015 and involved a total of 748 people (380 patients treated with BDP and 368 patients treated with 5-ASA). The average follow-up duration ranged from 4 to 8 weeks. The primary clinical outcomes were not consistent between the identified studies. Efficacy was evaluated by the DAI or other similar indexes. According to the specifics of each study, the authors formulated remission and clinical improvement criteria for statistical analysis. Among the included studies, only two articles evaluated the therapeutic effects of oral drugs [21,27]. The other five studies evaluated drugs administered by rectal enemas; therefore, we performed a subgroup analysis to compare BDP and 5-ASA using the same pharmaceutical preparation (oral or enema) [22–26].

**Table 1:** Characteristics of studies included.

	Year	Sample(^a^BDP/^b^5-ASA)	Female/male(n)	Mean age(years)	location of disease(n)	Intervention	Time evaluate	Country	Study design	method	Outcome	Evaluate method
Giochetti et al	2005	111/106	147/70	41.5	proctosigmoiditis 116	BDP(3mg/60ml^c^o.d)	6weeks [^d^DAI score]	Italy	^e^RCT	^f^ENE	clinical remission	remission: DAI score = 0
					Proctitis 62	5-ASA(1g/100ml o.d)					endoscopy	clinical improvement:DAI score reduced by at least 3 points
					Left-sided 39						side-effects	
L.Biancone et al	2007	50/42	58/34	^g^NR	proctitis NR	BDP(3mg/60ml)	4,8weeks[DAI score]	Italy	RCT	ENE	clinical remission	remission: DAI score<3
						5-ASA(1g/100ml o.d)					endoscopy	clinical improvement:DAI score reduced by at least 1 points
					proctosigmoiditis NR						side-effects	
M.Campieri et al	2003	90/87	107/70	43.25	left-sided 127	BDP(5mg/dayo.d)	4weeks[DAI score]	Italy	RCT	oral	clinical remission	remission: NR
					extensive 50	5-ASA(2.4g/day)					endoscopy	Clinical improvement:DAI score reduced by at least 3 points
											side-effects	
Franze et al	1999	37/34	NR	NR	left-side NR	BDP(3mg/60mlo.d)	4weeks[NR]	Italy	RCT	ENE	clinical remission	remission: NR
					proctitis NR	5-ASA(4g/60ml o.d)						
					proctosigmoiditis NR							
Mulder et al	1996	20/21	NR	41.34±14.04	Distal 20cm of the colon	BDP(3mg/100mlo.d)	4weeks[NR]	Netherland	RCT	ENE	clinical remission	remission: NR
						5-ASA(2g/100ml o.d)					endoscopy	
											Histological evaluation	
P.Crispino et al	2015	40/40	41/39	53±22.14	left-side 30	BDP(3mg/60ml)	8weeks[^h^CAI score]	Italy	RCT	ENE	clinical remission	remission: NR
					proctitis 22	5-ASA(4g/day)					side-effects	clinical improvement:NR
					proctosigmoiditis 28							
Pica R et al	2013	30/32	NR	NR	left-side 62	BDP(10mg/day)	8weeks[DAI score]	Italy	RCT	oral, ENE	clinical remission	remission: NR
						.5-ASA(4g/day)						clinical improvement:NR

^a^BDP: Beclomethasone dipropionate;

^b^5-ASA: Mesalazine;

^c^o.d: once day;

^d^DAI: Disease Activity Index;

^e^RCT: randomized controlled trials;

^f^ENE: enema;

^g^NR: not reported;

^h^CAI: Clinical Activity Index;

### Methodological assessment of study quality

**Fig 2:**
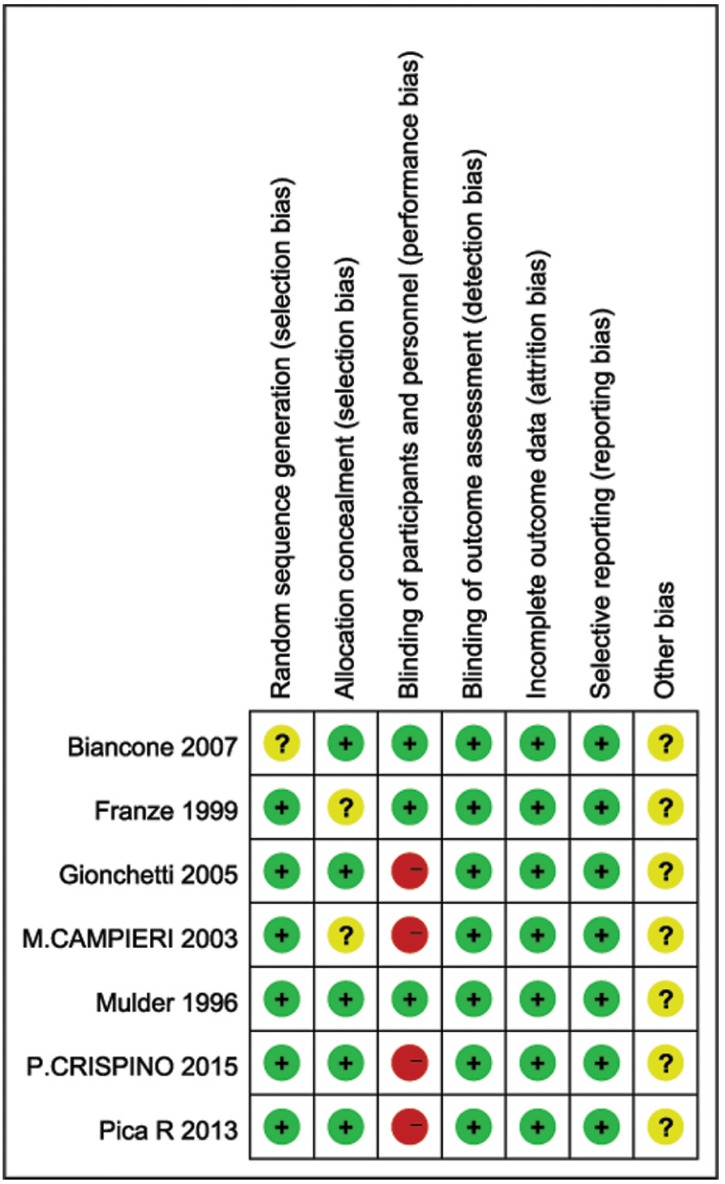
Risk of bias summary: This risk of bias tool incorporates the assessment of randomization (sequence generation and allocation concealment), blinding (participants and outcome assessors), incomplete outcome data, selective outcome reporting and other risk of bias. The items were judged as “low risk” “unclear risk” or “high risk”, red means “high risk”, green means “low risk” and yellow means “unclear risk”.

The methodological quality assessment of the 7 included studies is presented in Fig 2. The quality of these studies was low to moderate. Of the 7 RCTs, most studies clearly adopted random sequence generation using random number tables. Three studies [24–26] were double-blind, whereas the remaining four studies [21–23,27] were single-blind. Four studies [22–25] adopted adequate methods of allocation concealment, whereas the remaining three studies [21,26,27] did not describe concrete methods of concealment. Four studies [21–24] adopted adequate methods of randomization (e.g., computer-generated randomization schemes). Three studies that did not mention any information about their randomization methods were rated as unclear [24,26,27]. However, the method of randomization and the allocation concealment were not described in detail in some studies, which gave rise to high risks of selection and measurement bias. The quality of the tests conducted by such studies thus exhibited a high risk of selection bias, which may have affected the results.

### Remission and clinical improvement

**Fig 3:**
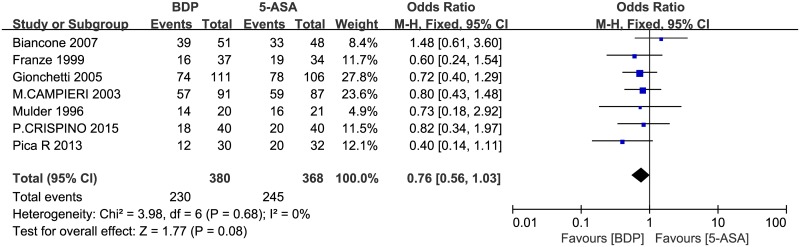
Forest plot of randomized controlled trials of BDP vs. 5-ASA in inducing remission and clinical improvement in ulcerative colitis. BDP, beclomethasone dipropionate. 5-ASA, mesalazine. M-H, Mantel-Haenszel.

From the aforementioned studies, to assess UC patients’ remission and clinical improvement, we identified a total of 748 UC patients: 380 patients in the BDP treatment group and 368 in the 5-ASA treatment group. Treatment times were 4 to 8 weeks. Efficacy was evaluated by the DAI or according to a study’s own formulated remission criteria for statistical analysis. Based on a summary of the data from each study, 230 (60.53%) patients in the BDP group achieved remission and clinical improvement, compared with 245 (66.58%) patients in the 5-ASA group. There was no heterogeneity between studies (P = 0.68, I^2^ = 0%), so we used the fixed-effects model to pool the data. The overall estimate indicated that the pooled OR was 0.76 (95% CI = 0.56–1.03, P = 0.08) and there is no obvious difference between the two groups with respect to remission induction and clinical improvement (Fig 3). Given that the upper confidence limit for the OR barely exceeds 1.0 and that the p-value is close to 0.05 for this primary efficacy outcome as well as that the horizontal block lies to the left of the vertical line, it indicates that the potential clinical efficacy of BDP may be better than 5-ASA.

### Subgroup analysis

**Fig 4:**
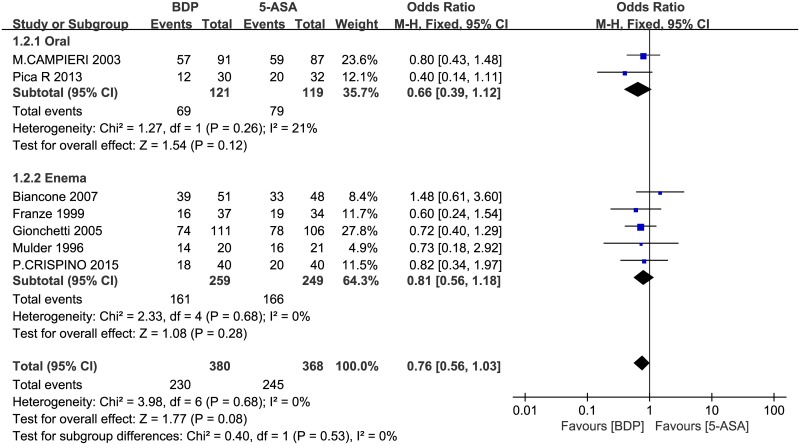
Forest plot of randomized controlled trials of BDP vs. 5-ASA with two pharmaceutical preparations (oral or enema) in inducing remission and clinical improvement in ulcerative colitis. BDP, beclomethasone dipropionate. 5-ASA, mesalazine. M-H, Mantel-Haenszel.

We then conducted a subgroup analysis to compare BDP and 5-ASA using the same pharmaceutical preparation (oral or enema). In the oral subgroup analysis, there was moderate heterogeneity between studies (P = 0.26, I^2^ = 21%), so we used the fixed-effects model to pool the data. The overall estimate indicated that the pooled OR was 0.66 (95% CI = 0.39–1.12, P = 0.12). In the subgroup analysis of studies on the effects of enema pharmaceutical preparations of the two drugs, no statistically significant heterogeneity was detected for this comparison (P = 0.68, I^2^ = 0%OR = 0.81, 95% CI = 0.56–1.18, P = 0.28). There is no obvious difference between the two groups with respect to remission induction and clinical improvement (Fig 4). Given that the upper confidence limit for the OR barely exceeds 1.0 and that the horizontal block lies to the left of the vertical line, it indicates that the clinical efficacy of BDP may be better than 5-ASA.

### Reduction of DAI parameters

For the assessment of UC patients’ remission and clinical improvement, the studies evaluated herein used the DAI or other similar grading systems. The DAI score is based on stool frequency, rectal bleeding, the physician-derived disease activity score, and intestinal mucosa quality. In three of the studies, assessment of the DAI scores after 4 or 6 weeks of treatment with the two drugs revealed significant DAI reductions [21,22,24]. A pooled analysis was performed for two of the studies, which had concrete DAI score values [21,22].

#### Stool frequency

**Fig 5:**
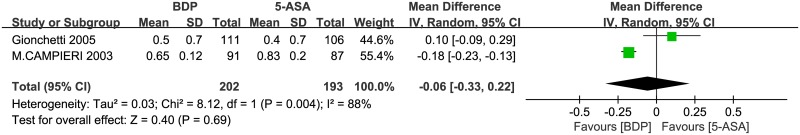
Forest plot of randomized controlled trials of BDP vs. 5-ASA in inducing Reduction of DAI parameters: stool frequency, in ulcerative colitis. BDP, beclomethasone dipropionate. 5-ASA, mesalazine. M-H, Mantel-Haenszel.

Stool frequency was compared between the two drug treatments. A heterogeneity test revealed significant heterogeneity among the studies (P = 0.004, I^2^ = 88%), so the random-effects model was used. A pooled analysis revealed that there was no significant difference between the BDP and 5-ASA groups (SMD = -0.06, 95% CI = -0.33–0.22, P = 0.69) (Fig 5).

#### Rectal bleeding

**Fig 6:**
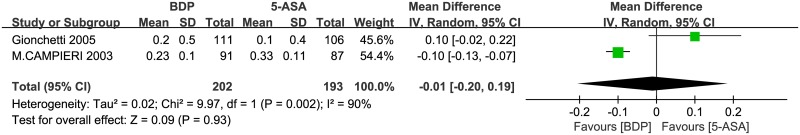
Forest plot of randomized controlled trials of BDP vs. 5-ASA in inducing Reduction of DAI parameters: rectal bleeding, in ulcerative colitis. BDP, beclomethasone dipropionate. 5-ASA, mesalazine. M-H, Mantel-Haenszel.

Rectal bleeding was compared between the two drug treatments. A heterogeneity test revealed significant heterogeneity among the studies (P = 0.002, I^2^ = 90%), so the random-effects model was used. A pooled analysis revealed that there was no significant difference between the BDP and 5-ASA groups (SMD = -0.01, 95% CI = -0.20–0.19, P = 0.93) (Fig 6).

#### Physician-derived disease activity scores

**Fig 7:**
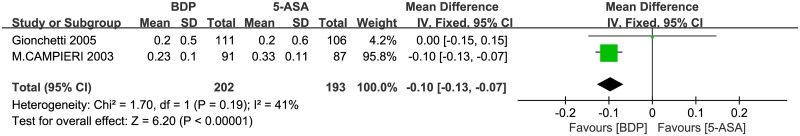
Forest plot of randomized controlled trials of BDP vs. 5-ASA in inducing Reduction of DAI parameters: physician-derived disease activity scores, in ulcerative colitis. BDP, beclomethasone dipropionate. 5-ASA, mesalazine. M-H, Mantel-Haenszel.

The physician-derived disease activity scores were compared between the two drug treatments. A heterogeneity test revealed moderate heterogeneity among the studies (P = 0.19, I^2^ = 41%), so the fixed-effects model was used. A pooled analysis suggested that the difference between the two groups was statistically significant (SMD = -0.10, 95% CI = -0.13 –-0.07, P < 0.00001) (Fig 7).

#### Intestinal mucosa

**Fig 8:**
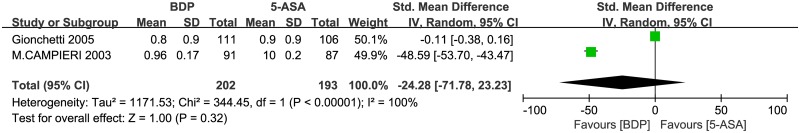
Forest plot of randomized controlled trials of BDP vs. 5-ASA in inducing Reduction of DAI parameters: intestinal mucosa, in ulcerative colitis. BDP, beclomethasone dipropionate. 5-ASA, mesalazine. M-H, Mantel-Haenszel.

Intestinal mucosa was compared between the two drugs. A heterogeneity test revealed significant heterogeneity among the studies (P < 0.00001, I^2^ = 100%), so the random-effects model was used. A pooled analysis revealed that there was no significant difference between the BDP and 5-ASA groups (SMD = -24.28, 95% CI = -71.78–23.23, P = 0.32) (Fig 8).

### Safety evaluation: complications and side-effects

**Fig 9:**
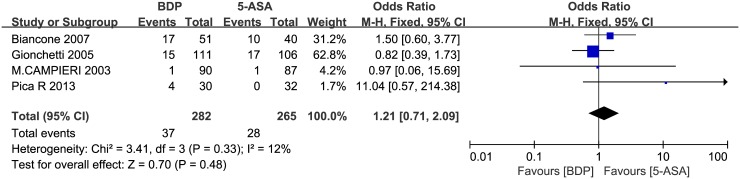
Forest plot of randomized controlled trials of BDP vs. 5-ASA in inducing complications and side-effects in ulcerative colitis. BDP, beclomethasone dipropionate. 5-ASA, mesalazine. M-H, Mantel-Haenszel.

Through a careful reading of the seven included studies, four were found to contain relevant data on patients with side effects, comprising a total of 547 patients; specifically, 37 out of 282 patients undergoing treatment with BDP reported adverse events, and 28 out of 265 patients undergoing treatment with 5-ASA showed side effects [21,22,24,27]. A pooled analysis indicated that there was no obvious difference in the incidence of complications and side-effects between two groups (OR = 1.21, 95% CI = 0.71–2.09, P = 0.48). (Fig 9). 17 patients received BDP, requiring drug discontinuation due to reported bloody or diarrhoea. Four patients of BDP group observed the reduction of mean plasma cortisol and had mild signs of hypothalamic-pituitary-adrenal axis (HPA) suppression. 10 patients received 5-ASA, requiring drug discontinuation due to reported abdominal pain or bowel tenderness. No clinically relevant change in laboratory parameters was observed in any study at the end of the treatment period. Taking into account that BDP has the risk of hypothalamic-pituitary-adrenal axis (HPA) suppression and that the horizontal block lies to the right of the vertical line, it indicates that the safety profile of 5-ASA may be better than BDP in the treatment of mild to moderate UC.

### Sensitivity analysis

Of the 7 studies, three studies that reported UC remission did not use DAI scores instead of Clinical Activity Index (CAI) scores or other similar criteria [23,25,26]. Therefore, we conducted a sensitivity analysis to determine whether the exclusion of these studies would change the effect estimate. Exclusion of these 3 studies from the meta-analysis did not substantially influence the summary estimate.

## Discussion

### Summary of main results

The development of 5-ASA and BDP topical formulations in recent years represents an important breakthrough in controlling the development of UC [28]. Although the efficacy of 5-ASA in UC treatment is significant, due to the larger and more frequent daily dose of 5-ASA required, patient's compliance is a challenge. However, once daily dosed formulation of 5-ASA is now available and improved patient's compliance [29]. BDP, a new second-generation glucocorticoid drug with low systemic bio-availability, has fewer side effects. Studies have shown that BDP at 3 mg/day and 5-ASA at 1–4 g/day (rectal administration) for the control of acute-phase distal UC have equal effects [22,24–26]. Although BDP has a satisfactory safety profile and clinical efficacy, its role in clinical practice is not yet well established.

In this study, we identified 7 RCTs investigating the efficacy, safety, and DAI scores associated with BDP and 5-ASA intervention. Our meta-analysis showed that BDP is as effective as 5-ASA in inducing remission and clinical improvement, thus demonstrating that in the treatment of UC, BDP and 5-ASA are equally efficacious. A subgroup analysis performed between different formulations of the drugs yielded the same results.

The decrease in DAI scores by BDP and 5-ASA interventions in the 7 included studies should also be discussed. These studies assessed UC patients’ remission and clinical improvement using the DAI or another similar grading system. DAI parameters included stool frequency, rectal bleeding, physician-derived disease activity score, and intestinal mucosa. Four studies assessed the DAI scores after 4 or 6 weeks of treatment with the two drugs and revealed significant decreases [21,22,24,27]. Only two studies had concrete values of DAI parameters [21,22]. A comparison of the data from these two studies showed that there was no difference between the two drug treatments with regard to stool frequency, rectal bleeding, and intestinal mucosa. However, there was a difference in the physician-derived disease activity scores (SMD = -0.10, 95% CI = -0.13 –-0.07, P < 0.00001). Our analysis shows that BDP use in the treatment of UC tends to get a low score in the physician's rating of disease activity, i.e., when treated with BDP, the disease goes into remission more easily.

The complications and side-effects noted in the 7 included studies should also be discussed.

One study reported that a patient developed serious pneumonia during the treatment with BDP. Ultimately, however, through investigation and judgment, the study concluded that the pneumonia that developed during the treatment of UC was not related to the study drug. In the BDP group, morning plasma cortisol levels remained unchanged compared with baseline values. During the treatment period, there were no clinically relevant changes in the laboratory parameters in either group [22]. In the literature, a detailed description of the specific side effects observed in 2007 noted the occurrence of bloody stool and diarrhea in BDP group; abdominal pain and bowel tenderness in 5-ASA group [24]. In Pica R’s study, safety was evaluated by monitoring adverse events, hematochemical parameters, and the study observed four patients in the BDP treatment group with mild signs of hypothalamic-pituitary-adrenal axis suppression [27]. In M. Campieri’s study, the incidence of adverse events was remarkably low in both treatment groups. Only two patients had adverse events; specifically, one patient in the BDP group reported menorrhagia, and one patient in the 5-ASA group developed influenza symptoms [21]. Although many patients reported adverse effects, most of these effects were relatively mild and of short duration. And no clinically revevant change in WBC, blood pressure, heart rate, body weight or other haematochemical parameters was observed in both groups. In summary, taking into account that the BDP has the risk of hypothalamic-pituitary-adrenal axis (HPA) suppression, 5-ASA has a potential advantage in the treatment of mild to moderate UC.

### Comparison with previous studies

In 2015, a recently completed systematic review of the treatment of UC supported the use of rectal therapies (corticosteroids and 5-ASA) for both induction and maintenance of remission in patients with distal forms of UC [30]. A study by Claudio Papi et al. enrolled 64 UC patients from January 2003 to December 2006 who were unresponsive to first-line treatment with 5-ASA; these patients received an 8-week course of oral BDP at a standard dose of 10 mg/day for 4 weeks and 5 mg/day for an additional 4 weeks. The study demonstrated that oral BDP could be a useful alternative to systemic corticosteroids as a second-line treatment for patients with UC who fail to respond to appropriate first-line treatment with 5-ASA [31]. After analysis, some of the studies included herein showed that the rectal administrations of BDP 3 mg/day and 5-ASA 1–4 g/day for the control of acute-phase distal UC have equal effects [22,24–26]. Another meta-analysis demonstrated that compared with conventional oral multidose (2 or 3 times daily) of 5-ASA, the once daily dosing of oral formulation of 5-ASA could improve patient's compliance and had equal efficacy in the treatment of mild to moderate UC [29].

To our knowledge, this is the first systematic review and meta-analysis comparing the efficacy and safety of BDP and 5-ASA for the treatment of UC performed with seven low- or moderate-quality RCTs. A meta-analysis published in 2007, which included only four RCT studies, found that BDP was equivalent to 5-ASA in inducing remission and clinical improvement [20]. However, the study was published nearly 8 years ago and identified only four RCTs comparing the two therapies. Moreover, that meta-analysis did not mention the side effects of the therapies. In our meta-analysis, we included the latest clinical RCTs of BDP and 5-ASA and excluded studies with a high risk of bias. To our knowledge, there has never been a comparably comprehensive and systematic review of UC treatment with BDP and 5-ASA to compare the safety and efficacy of the two drugs. To evaluate possible sources of heterogeneity, we also performed a subgroup analysis of different formulations and performed a sensitivity analysis to assess the influence of each study on the overall pooled estimate.

### Limitations of the study

Certain limitations of our meta-analysis should be described. First, causing that follow-up times of these included studies were less than 8 weeks, the comparison between BDP and 5-ASA mainly limited to induction treatment phase. Second, most patients had mild-moderate UC and a majority with only left sided disease, which limiting more wide applicability of these drugs; Third, some of the studies were of low quality and had relatively small sample sizes, thus the scarcity of high-quality, multi-center, large-sample standard RCTs which directly comparing these two drugs may influence the reliability of these results in this study. Fourth, the criteria for disease remission and recovery varied in each study. Moreover, there is no effective dose standard for treatment with 5-ASA and BDP, so there was a lack of uniformity of drug dosage and treatment duration among the various studies. Finally, in some studies, allocation of concealment was not reported, raising the issue of whether these trials were truly double-blind. These limitations, together with the fact that there were few trials making the comparisons of interest, mean that the true efficacy of these agents may not be as clear as the summary estimates from this meta-analysis would suggest.

## Conclusions

In summary, during induction treatment of mild to moderate UC, there is no obvious difference between the two groups with respect to remission and clinical improvement. Given that the upper confidence limit for the OR barely exceeds 1.0 and that the p-value is close to 0.05 for this primary efficacy outcome as well as that the horizontal block lies to the left of the vertical line, it indicates that the clinical efficacy of BDP may be better than 5-ASA. However, taking into account that BDP has the risk of hypothalamic-pituitary-adrenal axis (HPA) suppression, 5-ASA has a potential advantage of safety in the treatment of mild to moderate UC. Further studies, particularly longitudinal, high-quality, multi-center, large-sample standard RCTs to directly compare the two drugs, are warranted for further verification.

## Supporting Information

S1 File PRISMA Checklist. (DOC)

S2 File Excluded full-text articles. (DOCX)

## References

[1] Baumgart DC, Sandborn WJ. Inflammatory bowel disease:clinical aspects and established and evolving therapies. Lancet. 2007;369: 1641–1657. 17499606 10.1016/S0140-6736(07)60751-X

[2] Bamias G, Cominelli F. Immunopathogenesis of inflammatory boweldisease: current concepts. Curr Opin Gastroenterol. 2007;23: 365–369. 10.1097/MOG.0b013e3281c55eb2 17545770

[3] Ananthakrishnan AN. Environmental risk factors for inflammatory boweldiseases: a review. Dig Dis Sci. 2015;60: 290–298. 10.1007/s10620-014-3350-9 25204669 PMC4304948

[4] Kappelman MD, Moore KR, Allen JK, Cook SF. Recent trends in the prevalence of Crohn’s disease and ulcerative colitis in a commercially insuredUS population. Dig Dis Sci. 2013;58: 519–525. 10.1007/s10620-012-2371-5 22926499 PMC3576554

[5] Bernklev T, Jahnsen J, Aadland E, Sauar J, Schulz T, Lygren I, et al Health-related quality of life in patients with infl ammatory bowel disease five years after the initial diagnosis. Scand J Gastroenterol. 2004;39: 365–373. 10.1080/00365520310008386 15125469

[6] Kornbluth A, Sachar DB. Practice Parameters Committee of the American College of Gastroenterology. Ulcerative colitis practice guidelines in adults: American College Of Gastroenterology, Practice Parameters Committee Am J Gastroenterol. 2010;105: 501–23; quiz 524. 10.1038/ajg.2009.727 . 20068560

[7] Feagan B, MacDonald JK. Oral 5-aminosalicylic acid for induction of remission in ulcerative colitis. Cochrane Database Syst Rev. 2012;10: CD000543 10.1002/14651858.CD000543.pub3 23076889

[8] Rousseaux C, Lefebvre B, Dubuquoy L, Lefebvre P, Romano O, Auwerx J, et al Intestinal anti-inflammatory effect of 5-aminosalicylic acid is dependent on peroxisome proliferator-activated receptor-gamma. J Exp Med. 2005;201: 1205–1215. 10.1084/jem.20041948 15824083 PMC2213148

[9] Hench PS, Slocumb CH, Barnes AR, Smith HL, Polley HF, Kendall EC. The effects of the adrenal cortical hormone 17-hydroxy-11-dehydrocorticosterone (Compound E) on the acute phase of rheumatic fever; preliminary report. Mayo Clin Proc. 1949;24: 277–297. 18130001

[10] Truelove SC, Witts LJ. Cortisone in ulcerative colitis; preliminary report on a therapeutic trial. Br Med J. 1954;2: 375–378. 10.1136/bmj.2.4884.375 13182220 PMC2078989

[11] Hayashi R, Wada H, Ito K, Adcock IM. Effects of glucocorticoids on gene transcription. Eur J Pharmacol. 2004;500: 51–62. 10.1016/j.ejphar.2004.07.011 15464020

[12] Rutgeerts PJ. Review article: the limitations of corticosteroid therapy in Crohn’s disease. Aliment Pharmacol Ther. 2001;15: 1515–1525. 10.1046/j.1365-2036.2001.01060.x 11563990

[13] Bansky G, Bühler H, Stamm B, Häcki WH, Buchmann P, Müller J. Treatment of distal ulcerative colitis with beclomethasone enemas: high therapeutic efficacy without endocrine side effects. A prospective, randomized, double-blind trial. Dis Colon Rectum. 1987;30: 288–292. 10.1007/BF02556177 3030678

[14] Danese S, Fiocchi C. Ulcerative colitis. N Engl J Med. 2011;365: 1713–1725. 10.1056/NEJMra1102942 22047562

[15] Kumana CR, Seaton T, Meghji M, Castelli M, Benson R, Sivakumaran T. Beclomethasone dipropionate enemas for treating inflammatory bowel disease without producing Cushing’s syndrome or hypothalamic pituitary adrenal suppression. Lancet. 1982;1: 579–583. 6121181 10.1016/s0140-6736(82)91747-0

[16] Rizzello F, Gionchetti P, D’Arienzo A, Manguso F, Di Matteo G, Annese V, et al Oral beclometasone dipropionate in the treatment of active ulcerative colitis: a double-blind placebo-controlled study. Aliment Pharmacol Ther. 2002;16: 1109–1116. 10.1046/j.1365-2036.2002.01298.x 12030952

[17] Kumana CR, Seaton T, Meghji M, Castelli M, Benson R, Sivakumaran T. Beclomethasone dipropionate enemas for treating inflammatory bowel disease without producing Cushing’s syndrome or hypothalamic pituitary adrenal suppression. Lancet. 1982;1: 579–583. 6121181 10.1016/s0140-6736(82)91747-0

[18] Gionchetti P, Rizzello F, Habal F, Morselli C, Amadini C, Romagnoli R, et al Standard treatment of ulcerative colitis. Dig Dis. 2003;21: 157–167. 10.1159/000073247 14571113

[19] Manguso F, Balzano A. Meta-analysis: the efficacy of rectal beclomethasone dipropionate vs. 5-aminosalicylic acid in mild to moderate distal ulcerative colitis. Aliment Pharmacol Ther. 2007;26: 21–29. 10.1111/j.1365-2036.2007.03349.x 17555418

[20] Sutherland LR, Martin F, Greer S, Robinson M, Greenberger N, Saibil F, et al 5-Aminosalicylic acid enema in the treatment of distal ulcerative colitis, proctosigmoiditis, and proctitis.Gastroenterol. 1987;92: 1894–1898. 10.1016/0016-5085(87)90621-4 3569765

[21] Campieri M, Adamo S, Valpiani D, D’Arienzo A, D’Albasio G, Pitzalis M, et al Oral beclometasone dipropionate in the treatment of extensive and left-sided active ulcerative colitis: a multicentre randomised study. Aliment Pharmacol Ther. 2003;17: 1471–1480. 10.1046/j.1365-2036.2003.01609.x 12823149

[22] Gionchetti P, D’Arienzo A, Rizzello F, Manguso F, Maieron R, Lecis PE, et al Topical treatment of distal active ulcerative colitis with beclomethasone dipropionate or mesalamine: a single-blind randomized controlled trial. J Clin Gastroenterol. 2005;39: 291–297. 10.1097/01.mcg.0000155124.74548.61 15758622

[23] Crispino P, Pica R, Unim H, Rivera M, Cassieri C, Zippi M, et al Efficacy of mesalazine or beclomethasone dipropionate enema or their combination in patients with distal active ulcerative colitis. Eur Rev Med Pharmacol Sci. 2015;19: 2830–2837. 26241537

[24] Biancone L, Gionchetti P, Blanco Gdel V, Orlando A, Annese V, Papi C, et al Beclomethasone dipropionate versus mesalazine in distal ulcerative colitis: A multicenter, randomized, double-blind study. Dig Liver Dis. 2007;39: 329–337. 10.1016/j.dld.2007.01.012 17347061

[25] Mulder CJ, Fockens P, Meijer JW, van der Heide H, Wiltink EH, Tytgat GN. Beclomethasone dipropionate (3 mg) versus 5-aminosalicylic acid (2 g) versus the combination of both (3 mg/2 g) as retention enemas in active ulcerative proctitis. Eur J Gastroenterol Hepatol. 1996;8: 549–553. 10.1097/00042737-199606000-00010 8823568

[26] Franzè A, Galeazzi R, Marcucci F, Biraghi M. Topical treatment of ulcerative colitis. Doubleblind study between beclomethasone dipropionate and mesalazine. Minerva Gastroenterol Dietol. 1999;45: 287–296. 16498341

[27] Pica N, Unim H, Cassieri C. Oral beclomethasone dipropionate versus 5-aminosalycilic acid enema in active ulcerative colitis patients: lower efficacy but better compliance. Italian. J Med;64: 189–202. 18th Congresso Nazionale della Societa Scientifica FADOI Giardini Naxos Italy.2013,7:96.

[28] Kornbluth A, Sachar DB. Practice Parameters Committee of the American College of Gastroenterology. Ulcerative colitis practice guidelines in adults (update): American College of Gastroenterology, Practice Parameters Committee. Am J Gastroenterol. 2004;99: 1371–1385. 10.1111/j.1572-0241.2004.40036.x 15233681

[29] Feagan BG, MacDonald JK. Once daily oral mesalamine compared to conventional dosing for induction and maintenance of remission in ulcerative colitis: A systematic review and meta-analysis. Inflammatory Bowel Dis. 2012; 18: 1785–1794. 10.1002/ibd.23024 22644954

[30] Cohen RD, Dalal SR. Systematic Review: Rectal Therapies for the Treatment of Distal Forms of Ulcerative Colitis. Inflamm Bowel Dis. 2015;21: 1719–36. 10.1097/MIB.0000000000000379 26020604

[31] Papi C, Aratari A, Moretti A, et al Oral Beclomethasone Dipropionate as an Alternative to Systemic Steroids in Mild to Moderate Ulcerative Colitis Not Responding to Aminosalicylates. Digestive Diseases & Sciences. 2010;55: 2002–2007. 10.1007/s10620-009-0962-6 19937467

