# Supplementary material for: Efficacy and Safety of Beclomethasone Dipropionate versus 5-Aminosalicylic Acid in the Treatment of Ulcerative Colitis: A Systematic Review and Meta-Analysis
Source: PLoS One. 2016 Aug 8;11(8):e0160500. doi: 10.1371/journal.pone.0160500 (PMC4976912; doi:10.1371/journal.pone.0160500)
Supplement: S2 File — (DOCX) [file pone.0160500.s002.docx]

**Nine studies were excluded because they enrolled with other arms such as placebo or prednisone.**

1. Van Assche, G., et al., Corrigendum: Oral Prolonged Release Beclomethasone Dipropionate and Prednisone in the Treatment of Active Ulcerative Colitis: Results From a Double-Blind, Randomized, Parallel Group Study. Am J Gastroenterol, 2015. 110(6): p. 94

2. Van Assche, G., et al., Oral prolonged release beclomethasone dipropionate and prednisone in the treatment of active ulcerative colitis: results from a double-blind, randomized, parallel group study. Am J Gastroenterol, 2015. 110(5): p. 708-15.

3. Nunes, T., et al., Usefulness of oral beclometasone dipropionate in the treatment of active ulcerative colitis in clinical practice: the RECLICU Study. J Crohns Colitis, 2010. 4(6): p. 629-36.

4. Arcidiacono, R., et al.The topical therapy of ulcerative colitis. A multicenter study with beclomethasone dipropionate foam. Minerva Chir, 1999. 54(9): p. 635-44.

5. D'Arienzo, A., et al., Beclomethasone dipropionate (3 mg) enemas combined with oral 5-ASA (2.4 g) in the treatment of ulcerative colitis not responsive to oral 5-ASA alone. Ital J Gastroenterol Hepatol, 1998. 30(3): p. 254-7.

6. Campieri, M., et al., Beclomethasone dipropionate enemas versus prednisolone sodium phosphate enemas in the treatment of distal ulcerative colitis. Aliment Pharmacol Ther, 1998. 12(4): p. 361-6.

7. Halpern, Z., et al., A controlled trial of beclomethasone versus betamethasone enemas in distal ulcerative colitis. J Clin Gastroenterol, 1991. 13(1): p. 38-41.

8. Mulder, C.J., et al., Comparison of beclomethasone dipropionate (2 and 3 mg) and prednisolone sodium phosphate enemas (30 mg) in the treatment of ulcerative proctitis. An adrenocortical approach. Neth J Med, 1989. 35(1-2): p. 18-24.

9. van der Heide, H., et al., Comparison of beclomethasone dipropionate and prednisolone 21-phosphate enemas in the treatment of ulcerative proctitis. J Clin Gastroenterol, 1988. 10(2): p. 169-72.

**One citation study was excluded because it was a study about oral beclomethasone dipropionate in the treatment of ulcerative colitis.**

Rizzello, F., et al., Oral beclomethasone dipropionate in patients with mild to moderate ulcerative colitis: a dose-finding study. Adv Ther, 2001. 18(6): p. 261-71.

**One citation study was excluded because it added other medication.**

Rizzello, F., et al., Oral beclometasone dipropionate in the treatment of active ulcerative colitis: a double-blind placebo-controlled study. Aliment Pharmacol Ther, 2002. 16(6): p. 1109-16.

**One citation study was excluded because it was about the observation of beclomethasone dipropionate’s curative effect.**

Kumana, C.R., et al., Beclomethasone dipropionate enemas for treating inflammatory bowel disease without producing Cushing's syndrome or hypothalamic pituitary adrenal suppression. Lancet, 1982. 1(8272): p. 579-83.

**One citation study was excluded because the participants of this trial were children.**

Romano, C., et al., Oral Beclomethasone Dipropionate in Pediatric Active Ulcerative Colitis: A Comparison Trial With Mesalazine. Journal of Pediatric Gastroenterology and Nutrition, 2010: 50:385-389.
